# Weight change during chemotherapy changes the prognosis in non metastatic breast cancer for the worse

**DOI:** 10.1186/1471-2407-10-648

**Published:** 2010-11-25

**Authors:** Emilie Thivat, Sophie Thérondel, Olivier Lapirot, Catherine Abrial, Pierre Gimbergues, Emilie Gadéa, Eloïse Planchat, Fabrice Kwiatkowski, Marie A Mouret-Reynier, Philippe Chollet, Xavier Durando

**Affiliations:** 1 Division of Clinical Research, Centre Jean Perrin, Clermont-Ferrand, F-63011 France; University Clermont 1, UFR Médecine, Clermont-Ferrand, F-63001 France; Centre d'Investigation Clinique, Clermont-Ferrand, F-63001 France; 2 UMR 990 INSERM/UdA, Clermont-Ferrand, F-63000 France; 3 Oncology department, Centre Jean Perrin, Clermont-Ferrand, F-63011 France; University Clermont 1, UFR Médecine, Clermont-Ferrand, F-63001 France; Centre d'Investigation Clinique, Clermont-Ferrand, F-63001 France; 4 Surgery department, Centre Jean Perrin, Clermont-Ferrand, F-63011 France; University Clermont 1, UFR Médecine, Clermont-Ferrand, F-63001 France; Centre d'Investigation Clinique, Clermont-Ferrand, F-63001 France

## Abstract

**Background:**

Weight change during chemotherapy is reported to be associated with a worse prognosis in breast cancer patients, both with weight gain and weight loss. However, most studies were conducted prior to the common use of anthracycline-base chemotherapy and on North American populations with a mean BMI classified as overweight. Our study was aimed to evaluate the prognostic value of weight change during anthracycline-based chemotherapy on non metastatic breast cancer (European population) with a long term follow-up.

**Methods:**

Patients included 111 women diagnosed with early stage breast cancer and locally advanced breast cancer who have been treated by anthracycline-based chemotherapy regimen between 1976 and 1989. The relative percent weight variation (WV) between baseline and postchemotherapy treatment was calculated and categorized into either weight change (WV > 5%) or stable (WV < 5%). The median follow-up was 20.4 years [19.4 - 27.6]. Cox proportional hazard models were used to evaluate any potential association of weight change and known prognostic factors with the time to recurrence and overall survival.

**Results:**

Baseline BMI was 24.4 kg/m2 [17.1 - 40.5]. During chemotherapy treatment, 31% of patients presented a notable weight variation which was greater than 5% of their initial weight.

In multivariate analyses, weight change (> 5%) was positively associated with an increased risk of both recurrence (RR 2.28; 95% CI: 1.29-4.03) and death (RR 2.11; 95% CI: 1.21-3.66).

**Conclusions:**

Our results suggest that weight change during breast-cancer chemotherapy treatment may be related to poorer prognosis with higher reccurence and higher mortality in comparison to women who maintained their weight.

## Background

Age, tumour size, axillary node status, histological tumour type and standardized pathological grade are accepted as well-defined prognostic factors in breast cancer [1]. Various studies have also reported striking associations between overweight or obesity at breast cancer diagnosis and poorer prognosis with higher distant recurrence and mortality (for review [2]). As emphasized by Goodwin et al. [3], the risk of recurrence and death was respectively 1.78 (95% CI: 1.50-2.11) and 1.36 (95% CI: 1.19-1.55) times greater for obese patients over a 10 years follow-up period.

Moreover, numerous studies reported a weight gain after breast cancer development that might be attributable to the effects of some treatment regimens [4,5]. Weight gain in breast cancer patients has been associated with anti-neoplastic chemotherapy in the majority of studies. Previous studies suggest that weight gain is more pronounced among premenopausal women and among those who were treated with a multiagent regimen [6,7]. However, few reports have not observed increased weight gain during chemotherapy [5,8,9], in particular with anthracycline-containing regimens [10,11] widely recognized as the gold standard treatment of women with breast cancer.

There is also substantial evidence that weight change during chemotherapy may be associated with a worse prognosis for the cancer patient, both with weight gain [12] and weight loss [13]. The findings reported by the few studies which have explored the prognostic value of weight gain after a diagnosis of breast cancer are mixed: four studies reported that weight gain was associated with a decreased overall survival and increased recurrence risk [8,14-16] whereas five others failed to report such associations [10,9,17]. Only one recent study reported some evidence that women with early breast cancer, who had a weight loss during treatment, were at higher risk of recurrence and death compared to women with no weight variation [18]. These discrepancies may be attributable to the heterogeneity of the methods implemented in the different studies, including the duration of post-diagnosis weight assessment, the definition of the prognostic outcomes with a short median of follow-up and treatment (chemotherapy and/or hormonotherapy...). The majority of the previous studies were conducted before anthracycline-base chemotherapy was commonly used. Additionally all the aforementioned studies focused on north American populations with a mean Body Mass Index (BMI) at breast cancer diagnosis classified as overweight [14,19]. However no data is available on the prognostic impact of weight change during chemotherapy treatment in European breast cancer patients who presented a notably lower BMI [20].

Our study thus investigated the prognostic value (death and recurrence) of weight variation during anthracycline-based chemotherapy treatment of breast cancer in a French population with a long-term follow-up. We also verified the association of weight at breast cancer diagnosis with survival.

## Methods

### Population

A retrospective chart review was performed using data from hospital medical records on all women with early stage breast cancer and locally advanced breast cancer who were treated at Jean Perrin Center (Clermont-Ferrand) between 1976 and 1989 in order to have at least 20 years of follow-up for the study. Among the 709 women treated with chemotherapy treatment for breast cancer, 111 women were included in the analysis. The study was approved by the Inter-regional Ethics Committee of the Rhône -Alpes-Auvergne Clinical Investigation Center (N°IRB5044). Subjects were selected if they had histologically confirmed stage I-III breast cancer, and received chemotherapy under the anthracycline-based chemotherapy regimen. Patients were excluded from this review if weight assessment did not include at least measurements at baseline and at the end of chemotherapy, if they had distant metastasis at diagnosis or a history of another malignancy.

### Weight measurements

Weight was measured at the hospital by a nurse at the beginning of treatment and in the last chemotherapy cycle. The BMI was calculated by dividing weight (kg) by height (m) squared. The different subclasses of patients were categorized as followed: underweight (< 18.5), normal (18.5 - 25), overweight (25 - 29.9) or obese (≥ 30). The median BMI being of 24.4 kg/m^2 ^rounded down to 24 kg/m^2 ^was used to median-split the population, i.e. categorized women as having BMI less than 24, or more than 24 kg/m^2^.

A number of studies suggest that a 5% change in body weight is clinically meaningful [5]. Weight variations (WV) were calculated as the relative percent weight changes between weight measurement from baseline to post-chemotherapy treatment ((baseline weight - weight after chemotherapy)/baseline weight × 100). WV were categorized accordingly into weight change (WV > 5%) or stable (WV < 5%). The weight changing group (WV > 5%) combined women who lost weight (defined by a relative weight loss >5% between weight measurement from baseline to post-chemotherapy treatment) and women who gained weight (defined by a relative weight gain > 5%).

### Covariates

Information on the age of patients, menopausal status, hormonal receptors, tumour stage, nodal involvement, Scarff-Bloom-Richardson (SBR) grade, and on treatment received before and after chemotherapy were obtained from reviewing patients' medical records. We used the Tumour- Node-Metastasis (TNM) classification of stage of breast cancer at diagnosis as established by the American Joint Committee on Breast Cancer [21] which consists of 3 components: (i) tumour size (T); (ii) absence or presence and extent of regional lymph node metastasis (N); and (iii) absence or presence of distant metastasis (M).

### Outcome assessment

Deaths and recurrences were last updated in June 2009. Recurrence included a local/regional cancer recurrence, distant recurrence/metastasis, or development of a contralateral primary breast cancer. Patients who died without recurrence of breast cancer beforehand have been censored for analysis of recurrence.

The disease free survival (DFS) duration was defined as the time elapsed between the date of first diagnosis and the date of first relapse. The overall survival (OS) duration was the time elapsed between the date of initial diagnosis and the date of death or the last status report, whether the patient was alive or dead, whatever the cause.

### Statistical analyses

Descriptive statistics were calculated for all variables used in this study and presented as median [range]. OS and DFS were estimated using Kaplan-Meier method [22]. A univariate analysis was performed using log rank methods. Parameters tested to be potentially correlated with OS or DFS were BMI and WV.

We realized multivariate analysis using Cox's proportional-hazard models [23] to evaluate the association of categories of baseline BMI and WV and well defined pronostic factors in breast cancer with the time to recurrence and mortality. Covariates considered as potential confounders in the above model included menopausal status, tumour stage, nodal involvement, and treatment after chemotherapy (hormonotherapy). A *p *value < 0.05 was considered to be statistically significant. Variance analysis (Chi^2 ^or Kruskal-Wallis H tests) was used to test associations between initial BMI, WW and the covariates. Analyses were conducted using SEM software version 3.5 [24].

## Results

### Characteristics of the population

Table 1 lists the main characteristics of patients. The median age at diagnosis was 54 years (32 - 55 years), and 55% of the women were post-menopausal at diagnosis. In all, 58 patients (52%) had a positive hormone receptor status and among them 28 were ER+/PR+ (25%). Seventeen percent of the women were diagnosed with stage I breast cancer while 48% had stage II and 35% had stage III respectively. With regards to tumour characteristics, 21 patients (19%) were T1, 49 T2 (44%), 17 T3 (15%) and 24 T4 (22%), respectively. Regarding clinical node involvement, 55 patients were N0 (50%), 49 N1 (44%), 6 N2 (5%) and 1 N3 (1%), respectively.

**Table 1:** Main characteristics of the population study

**Characteristics, n = 111**		**stable weight** **(n = 77)**	**changing weight** **(n = 34)**	***P***
	
Median age (years (range))	54 (32 - 74)	53 (37 - 70)	54 (32 - 74)	*0,49*
	
	n (%)			
	
Median BMI	24.4 (17.1 - 40.5)	24.6 (17.1 - 40.5)	24.4 (16.6 - 40.5)	*0,14*
underweight	9 (8)	7 (9)	2 (6)	
normal	56 (50)	36 (47)	20 (59)	
overweight	31 (28)	21 (27)	10 (29)	
obese	15 (14)	13 (17)	2 (6)	
	
Menopausal status				*0,78*
Premenopausal	50 (45)	34 (44)	16 (47)	
Menopausal	61 (55)	43 (56)	18 (53)	
	
Oestrogene receptors				*0,61*
Positive	47 (42)	32 (42)	15 (44)	
Negative	48 (44)	35 (45)	13 (38)	
	
Progesteron receptors				*0,73*
Positive	39 (35)	29 (38)	10 (29)	
Negative	52 (47)	37 (48)	15 (44)	
	
Tumor stage				*0,99*
T_1_	21 (19)	15 (19)	6 (18)	
T_2_	49 (44)	34 (44)	15 (44)	
T_3_	17 (15)	12 (16)	5 (18)	
T_4_	24 (22)	16 (21)	8 (24)	
	
Clinical node involvement				*0,34*
N_0_	55 (50)	34 (44)	21 (62)	
N_1_	49 (44)	37 (48)	12 (35)	
N_2_	6 (5)	5 (6)	1 (3)	
N_3_	1 (1)	1 (1)		
	
SBR grade				*0,17*
I	9 (8)	8 (10)	1 (3)	
II	61 (55)	40 (52)	21 (62)	
III	22 (20)	8 (10)	4 (12)	

### Treatments

Between 1976 and 1989, patients received a median number of 6 cycles [2-15] of polychemotherapy. The median lag time between diagnosis and the start of treatment was 1.7 months [0-9]. All patients were treated with an anthracycline-based regimen (AVCF 54%, AVCFM 44%, FAC 1%, FEC 1%). In all, 66 patients underwent a tumourectomy and 44 underwent a mastectomy. After chemotherapy, 97% received radiation and 44% a hormonal therapy (90% with tamoxifen).

### BMI and weight variation

The initial median BMI was 24.4 kg/m2 [17.1 - 40.5 kg/m2]. The different subclasses of patients were distributed as followed: 9% were underweight, 56% normal, 31% overweight and 15% obese.

During chemotherapy, weight was stable with a median relative WV of 0 [-10.9 - 15.4%]. Using a threshold of 5%, 17% of patients lost weight, 69% were stable and 14% gained weight. Thus, 31% of patients presented a notable WV, higher than 5%.

### Univariate analyses

The median of the follow-up was 20.4 years [19.4 - 27.6]. Among the 111 women, 57 died, 14 developed a local recurrent, and 56 a distant metastasis. Only few patients died from other causes than breast cancer. Among the 57 women who died, 47 developed breast cancer recurrence (83%). No patient was lost of follow-up. The median OS was 14.3 years [0.7 - 21.8] and median DFS 10 years [0.4 - 21]. The univariate analysis sh owed that OS (*p *= 0.002) and DFS (*p *= 0.0039) depended on tumour stage. We also found that nodal involvement influenced OS (*p *< 0.001) and DFS (*p *= 0.0024).

**Figure 1:**
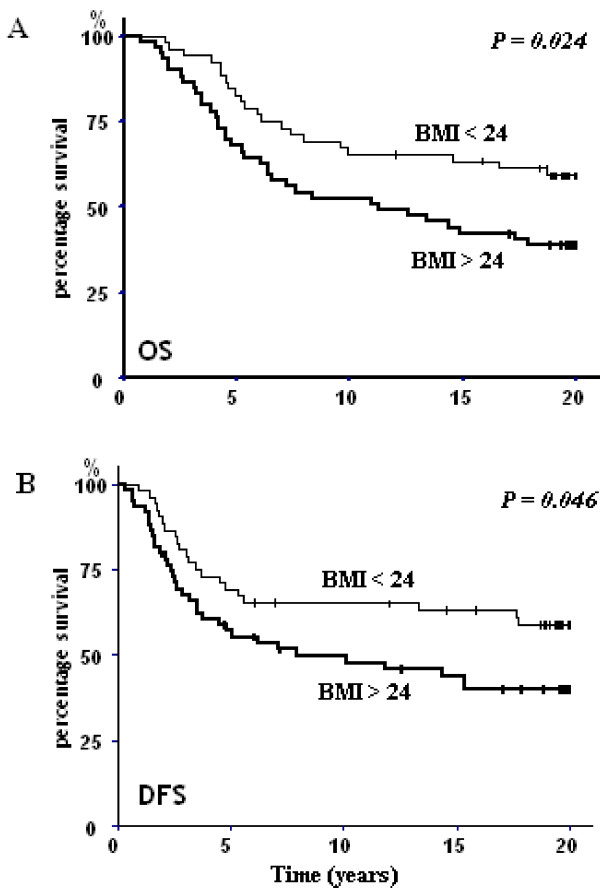
**Kaplan-Meier overall survival (OS) (A) and desease-free survival (DFS) (B) of patients whom initial BMI was < 24 kg/m^2 ^(BMI < 24) and > 24 kg/m^2 ^(BMI > 24)**.

Concerning the initial BMI, patients with a baseline BMI of less than 24 kg/m^2 ^had a better OS than those with an initial BMI of greater than 24 kg/m^2 ^(*p *= 0.024; Figure 1A). DFS was also influenced by the BMI, as illustrated by a significant statistical difference between these two groups (*p *= 0.046; Figure 1B).

**Figure 2:**
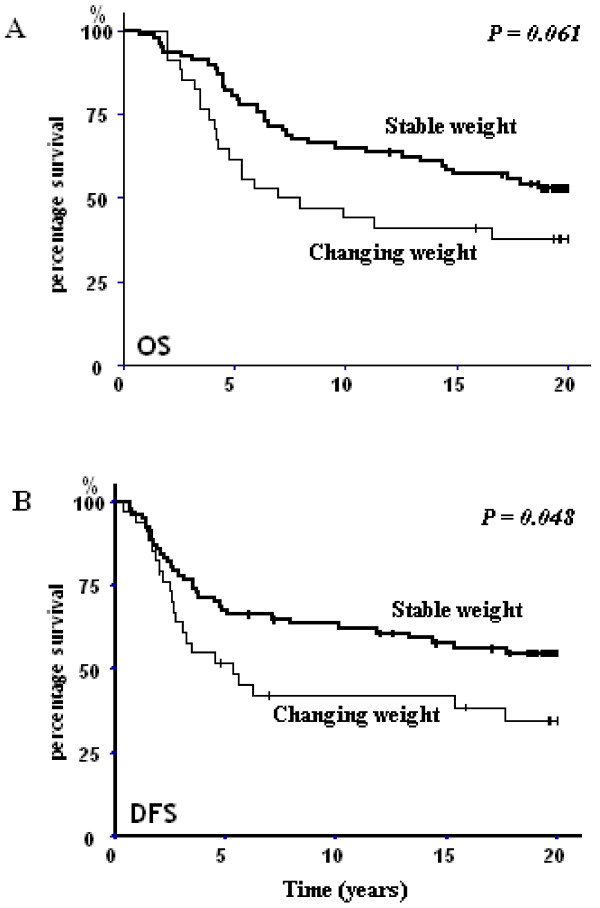
**Kaplan-Meier overall survival (OS) (A) and desease-free survival (DFS) (B) of patients whom weight variation was < 5% (stable) and > 5% (changing weight)**.

Moreover, OS and DFS were influenced by the WV. As the sample size is likely too small to detect significant effects of weight gain or loss as independent factors, we therefore chose to group women who gained weight with those who lost weight as a weight changing group compared to women with no weight variation. Indeed, a Kaplan-Meier analysis revealed a significant DFS difference between patients whose weight varied beyond 5% compared to patients who maintained their weight (*p *= 0.048; Figure 2B) while OS analysis was closed to significance (*p *= 0.061; Figure 2A).

Variance analysis revealed a significant correlation between initial BMI and tumour stage (*p *= 0.0038), menopausal status (*p *= 0.00074) but not with clinical node involvement, hormonotherapy treatment, and WW. Results from the Chi^2 ^test showed no significant correlation between weight variation and tumour stage, nodal involvement, menopausal status, initial BMI and administration of hormonotherapy after chemotherapy.

### Multivariate analysis

The multivariate Cox model (Table 2) included WV, tumour stage, nodal involvement, initial BMI, menopausal status and treatment by hormonotherapy. As mostly patients received radiotherapy (97%), this factor was not included in multivariate analysis.

**Table 2:** Multivariate Cox model for overall survival (OS) and desease-free survival (DFS)

Category	OS				DFS			
	*e/n*	RR	95% - CI	P	*e/n*	RR	95% - CI	P
Weight variation				0.0082*				0.0046*
< 5% (reference)	*36/77*	1.00			*34/77*	1.00		
> 5%	*21/34*	2.11	1.21 - 3.66		*21/34*	2.28	1.29 - 4.03	
								
Clinical Node Involement				0.054				0.021*
N0 (reference)	*22/55*	1.00			*23/55*	1.00		
N1	*30/49*	1.61	0.99 - 2.61		*27/49*	1.78	1.09 - 2.91	
N2	*4/6*	2.59	0.98 - 6.80		*4/6*	3.18	1.19 - 8.49	
N3	*1/1*	4.16	0.99 - 16.40		*1/1*	5.66	1.29 - 24.76	
								
Tumor stage				0.084				0.14
T1 (reference)	*6/21*	1.00			*7/21*	1.00		
T2	*24/49*	1.32	0.96 - 1.81		*25/49*	1.17	0.85 - 1.60	
T3	*10/17*	1.74	0.93 - 3.27		*7/17*	1.37	0.73 - 2.57	
T4	*17/24*	2.30	0.90 - 5.90		*16/24*	1.60	0.60 - 4.13	
								
Initial BMI				0.20				0.33
< 24 kg/m2	*21/52*	1.00			*21/52*	1.00		
≥ 24 kg/m2	*36/59*	1.49	0.81 - 2.74		*34/59*	1.59	0.86 - 2.93	
								
Menopausal status				0.79				0.46
Premenopausal (reference)	*21/50*	1.00			*23/50*	1.00		
Menopausal	*36/61*	1.09	0.59 - 1.99		*32/61*	0.96	0.53 - 1.76	
								
Hormonotherapy				0.81				0.90
no (reference)	*34/67*	1.00			*36/67*	1.00		
yes	*23/44*	0.93	0.53 - 1.65		*19/14*	0.80	0.44 - 1.14	

* P value considered statistically significant (p < 0.05).

All the variable in the table were mutually adjusted for each other.

Only WV still had a significant effect on OS. A change of weight of more than 5% was associated with an increased risk of death of 2.11; 95% CI: 1.21-3.66 (*p *= 0.0082).

Moreover, we found that WV was associated with a risk of recurrence of 2.28; 95% CI: 1.29-4.03 (*p *= 0.0046). Although DFS was significantly affected by clinical node involvement (*p *= 0.021). The tumour stage, initial BMI, menopausal status and treatment by hormonotherapy having a significant influence on DFS or on OS was not observed.

## Discussion

In this study, we demonstrate a relationship between weight variation during polychemotherapy treatment and both poorer disease-free survival and overall survival after diagnosis of breast cancer.

The present study is in agreement with previous studies which have found that overweight at the time of diagnosis increased both breast cancer recurrence and mortality. This result has been largely demonstrated in U.S. populations [25-27]. The poorer prognosis of obesity at diagnosis could be explained by the fact that overweight women tend to be diagnosed with later stage cancer and therefore more adverse tumour characteristics than normal weight women [28]. For some authors, this association is restricted to women who detected their own cancer and not spread amongst cases detected by either screening mammography or clinical breast examination [29]. Additionally, lower screening rates may partly explain the higher breast cancer mortality in obese women [30]. In agreement with this, in our population who were diagnosed 20 years ago, when no screening mammography was systematically carried out, we observed a striking association between BMI and tumour size.

In our series however, with a median WV equal to zero during chemotherapy treatment of breast cancer patients, 31% of our population presented a significant weight variation (> 5%) whereas 68% had maintained their weight. Our findings of no significant median WV during chemotherapy treatment are in contrast with the vast majority of studies conducted in North America, which have generally reported an average weight gain ranging from 1.7 to 4.4 kg during the years that follow diagnosis in women treated by chemotherapy [5,31,32]. On the other hand, one study carried out in a Korean breast cancer population has already observed a lack of overall weight gain, with 10.4% of the population gaining more than 5% of baseline body weight at 1 year [31]. Regarding the baseline mean BMI, we can observe that populations who did not display a significant weight gain during chemotherapy treatment, including ours, are leaner (mean BMI of 24.4 kg/m2 in our study, 23.5 kg/m2 in Korean study) than the ones used in the U.S. studies [5] in which the mean BMI varied from to 26.3 to 27.4 kg/m2 [18,32]. Moreover, one possible explanation is that the chemotherapy regimen administered to our patients incorporated anthracycline-based therapy as reported by Han and al. [33], whereas the majority of the earlier previous studies that observed a weight gain, involved non-anthracycline-based regimen. Other retrospective and prospective reports have not demonstrated increased weight gain with anthracycline-containing regimens compared with other regimens [10,11]. Fisher et al. [34] noted that 14.4% of patients receiving treatment with the AC regimen gained ≥ 5% over pretreatment weight compared with 42.2% of patient receiving CMF. This result was comparable to our result (14% gained weight) indeed the fact that patient accrual took place prior to the widespread use of 5-HT_3 _receptor antagonists in the two studies (cancer-related treatment currently used to reduce the impact of nausea and emesis associated with anthracycline use).

The long-term follow-up of patients who received an anthracycline-based chemotherapy in this study demonstrates that weight variation may not only influence recurrence, but also patient outcome. In multivariate analysis, clinical node involvement was still significant, thus, weight change was the strongest parameter associated with OS and DFS in our series. Literature on prognostic value of weight variation is mixed and not easily comparable. These studies that generally evaluated post-diagnosis weight variation on different periods which varied from a few months to a few years after diagnosis, including different treatments (chemotherapy and or hormonotherapy, radiation only...), did not use the same prognostic outcomes, and sometimes with a short median of follow-up. Among the few studies which evaluated the prognostic value of weight change after breast cancer diagnosis, four studies have shown a poor prognosis [8,14-16] whereas five reported no relationship [9,17-19].

The majority of studies observed an impact of weight gain on patient outcome. The largest study to date included 5,204 Nurses' Health Study participants diagnosed with non-metastatic breast cancer between 1976 and 2000 treated with chemotherapy and/or hormonal therapy [14]. This study reported an increased risk of recurrence, breast cancer death and total mortality in patients who gained more than 2 kg/m^2 ^by comparison to patients who maintained their weight. However this relationship was found only among women who never smoked and the definition of recurrence included reported lung, bone or brain cancer, but excluded any local recurrences in the ipsilateral breast or new primaries in the contralateral breast. Camoriano et al. reported weight gain having a significant effect on overall survival during treatment with cyclophosphamide, fluorouracile and prednisolone (CFD) or CFD plus tamoxifen, but not on recurrence and this only for premenopausal women [8]. Two other studies conducted prior the common use of anthracycline-based chemotherapy reported a correlation between weight gain and overall survival and/or disease-free survival (without defining which events were included) [15,16].

Only one recent study reported some evidence that women with early stage breast cancer treated with chemotherapy and/or radiation and tamoxifen who had large weight loss (> 10%) were at higher risk of recurrence and death compared to women with no weight variation. This elevated risk was more pronounced among women who were obese before diagnosis or who had ER- or PR- tumours [19]. One obvious explanation for significant weight loss being related to an increased risk of death could be that the breast cancer disease process itself caused weight loss.

**Figure 3:**
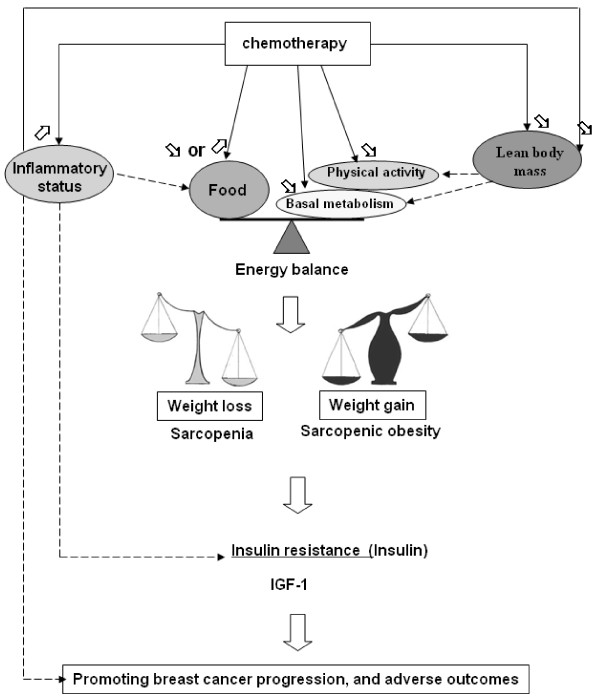
**Possible mechanisms to explain weight change during chemotherapy treatment of non metastatic breast cancer and its prognostic value**.

Literature on the prognostic value of weight variation reported some evidence that women who had gained or lost weight have a higher risk of recurrence and death compared to women with no weight variation. So, we chose to group women who gained weight with those who lost weight as a weight changing group. We hypothesized that weight change reflected a metabolic disorder by comparison to women who maintained their weight with an energy balance in equilibrium (Figure 3). Chemotherapy induced a decrease in energy expenditure (lowered basal metabolic rate, thermogenesis, and physical activity [4]) and different modifications in dietary intake (increase in appetite [14] or decreased ingestion of food due to chemotherapy related nausea and emesis) that can lead to weight gain or loss according to dietary behaviour of patient. Moreover, women with breast cancer receiving adjuvant chemotherapy underwent unfavourable changes in body composition with lean body loss due to a negative nitrogen balance [35] even in the absence of an overall weight change [4]. A lot of data has demonstrated that weight gain during chemotherapy was indicative of sarcopenic obesity [33]. Indeed, chemotherapy for breast cancer like taxane and anthracycline can increase inflammation [36,37] which played a central role on different modifications induced by chemotherapy. Inflammatory cytokines interfered with the satiety centre [38] and catabolism of skeletal muscle protein responsive of sarcopenia but not independently of any of the considered obesity indexes [39].

Several mechanisms have been proposed to explain the adverse effect of weight gain on risk of recurrence and mortality. First, weight gain and all associated metabolic disorders may predispose women to diabetes or heart disease, thereby predisposing them to morbidity and mortality [6]. However, in our study, only a few patients died from causes other than breast cancer (14%). One possible mechanism was a greater aromatase activity in the excess adipose tissue [40] and an inhibition of synthesis of sex hormone-binding globuline associated with an increased in free estradiol level which stimulates neoplasic cells [41].

Insulin resistance may be a common mechanism to explain the poor prognosis of patients who experienced a weight loss or a weight gain (Figure 3). Indeed, insulin resistance has been shown in variety of cancer patients with body-weight loss [42], but was also seen in overweight women [43]. Fasting serum insulin concentration has been directly associated with an increase in both distant recurrence and death in women previously treated for breast cancer [42,43]. There is a strong biological rational for an adverse prognostic effect of insulin. Insulin, a member of a family of growth factors that includes IGF-I and IGF-II, exerts a mitogenic effect on malignant breast cancer cells though IGF-I receptor. It is also hypothesized that visceral obesity increases both insulin-like growth factors (IGF-I, IGF-II) which stimulates the synthesis of sex steroid hormones [31] that are involved in the regulation of normal and malignant growth of epithelial breast cells. Several studies have reported a reduction in circulating concentration of IGF-I in malignant disease, which may also have been associated with nutritional decline and systemic inflammation [44]. Yoshikawa et al. hypothesized that inflammatory reactions might be involved in the development of insulin resistance [42]. Moreover, few studies reported an association between elevated inflammatory cytokines and a worse prognosis in breast cancer patients [45,46].

Further research is needed to understand the biological mechanisms underlying the relationship between weight variation and breast cancer growth with exploration of insulin resistance in association with body composition, measurement of energy expenditures, calorie intake and inflammatory reaction.

The current study had several limitations including its study design (retrospective chart review) and a relatively small sample size to draw a conclusion on the independent effect of weight variation. Some interesting covariates like "normal " weight prior diagnosis, smoking status, physical activity or sociodemographic aspects (education) have not been explored because of the lack of this data in patients' medical records. Additionally, more detailed measurement of body shape and fat content are lacking. Currently a long-term measurement of weight during patient follow-up is ongoing and could offer the possibility to explore weight variation after treatment which could also affect prognostic outcomes.

## Conclusions

Our results suggest that weight change during anthracycline-based treatment of early stage breast cancer is associated with increased risk of recurrence and poorer survival, though they may require additional confirmation. Furthermore, while we have speculated on potential biological targets, more research is needed to understand the biological mechanisms underlying the relationship between weight variation and breast cancer growth.

## Abbreviations

WV: weight variation; BMI: Body Mass Index; TNM: tumor-node-metastasis; DFS: disease free survival; OS: overall survival; CI: confidence Interval.

## Competing interests

The authors declare that they have no competing interests.

## Authors' contributions

ET contributed to the data collection, analysis and wrote the paper. ST and OL contributed to the data collection and helped in in writing the final draft. FK contributed to the data analysis. EG, CA, EP helped in writing the final draft. PG, MAMR, PC and XD contributed to patient recruitment. PC and XD contributed to the study design and analysis and have been involved in drafting the manuscript. All authors read and approved the final manuscript.

## Pre-publication history

The pre-publication history for this paper can be accessed here:

http://www.biomedcentral.com/1471-2407/10/648/prepub
